# An EEG Study of the Influence of Target Appearing in the Upper and Lower Visual Fields on Brain Attention Resource Allocation

**DOI:** 10.3390/brainsci13030433

**Published:** 2023-03-02

**Authors:** Han Li, Jialin Xu, Junpeng Sheng, Huilin Zhou, Zhen Liu, Yahui Li, Jingyan Hu, Guokun Zuo

**Affiliations:** 1 Faculty of Information Science and Technology, Ningbo University, Ningbo 315211, China; 2 Cixi Institute of Biomedical Engineering, Ningbo Institute of Materials Technology and Engineering, Chinese Academy of Sciences, Ningbo 315300, China; 3 Ningbo College of Materials Engineering, University of Chinese Academy of Sciences, Ningbo 315201, China

**Keywords:** event-related potentials, EEG rhythms, attentional resources, upper and lower visual fields, ERD/ERS

## Abstract

Visuospatial selective attention can focus attention on a certain spatial area and rationally allocate attentional resources during visual target perception. Previous studies investigated the behavioral advantages of subjects when the target appeared in the upper and lower visual fields. However, the neurophysiological characteristics of the brain are not clear, and there is a lack of comprehensive analysis of the external behavior and the internal neurophysiological characteristics. We designed two task paradigms containing a spatial location orientation task and a visual search task. We used event-related potentials (ERP) components (P1 and P2) and electroencephalogram (EEG) rhythms (theta and alpha) to analyze the attention level and allocation of attention resources of the brain. The results showed that when the target appeared in the lower visual field in the spatial location orientation task, subjects consumed fewer attention resources and demonstrated better behavioral performance. In the visual search task, when the target appeared in the upper left visual field, subjects could better mobilize attention resources and behaved more advantageously. The study provides a basis for the design of the target in the upper and lower visual fields in the rehabilitation task, especially for stroke patients with low attention levels due to attention disorders such as spatial attention deficit.

## 1. Introduction

As one of the most important cognitive functions of humans, visuospatial selective attention can focus attention on a certain spatial area and allocate attentional resources rationally during the perception of the visual target. The “spotlight theory” of attention also suggests that attention, like a spotlight, can be “focused” on a region of visual space at any given moment, and only stimuli in this area can be effectively perceptually analyzed [1,2]. However, most stroke patients have spatial attention disorder, so their spatial attention distribution and spatial attention fineness will be affected. The patients with the above conditions are characterized by decreased attention to visual stimuli [3] and are more likely to ignore the details of spatial stimuli [4], which is related to the abnormality of the ventral–dorsal attention pathway in stroke patients [5]. The target-oriented rehabilitation task training can improve cognitive and limb-motor functions in stroke patients, which requires the involvement of attention [6]. So, how can we design the spatial information of the target in rehabilitation tasks to help the patients mentioned above with spatial attention disorder to better perceive and process the target? This will facilitate their further rehabilitation training.

The upper and lower visual fields are the basis of human visual function. The receiving and processing of stimuli in the upper and lower visual fields reflect the perception of visual information (visual perception) and brain processing of visual information (visual processing), which is asymmetric. Our study focused on the influence of a target that appeared in the upper and lower visual fields on brain attention resource allocation. As we all know, in brain anatomy, the visual information of the upper and lower visual fields is projected to the ventral and dorsal sides of the brain through cross-mapping, and the visual information is transmitted through the ventral and dorsal pathways [7]. Behaviorally, the dominance of the upper and lower visual fields has been found in different tasks. Among them, the dominance of the lower visual field has been found in more tasks. For example, illusory contour perception task [8], visually guided perception task [9], and spatial reorientation memory task [10], etc. A few studies have found the dominance of the upper visual field, such as the visual search task [11] and the sweeping eye movement task [12,13]. These studies concluded that the task benefits were reflected in behavioral performance, such as higher response accuracy and faster reaction time. Furthermore, some studies also investigated the influence of visual effects on subjects’ behavior. For example, they manipulated the spatial frequency [14] and eccentricity [15,16] of the target stimulation. They considered that the asymmetry of the upper and lower visual fields was affected by different visual factors, and the difference in behavior performance might reflect different visual processing efficiency. Few studies have investigated the dominance of the upper and lower visual fields at the neurophysiological level. Several studies used event-related potentials (ERP) components P1 and P2, and electroencephalogram (EEG) rhythms theta and alpha to analyze the brain’s attention level and attention resource allocation in non-upper and lower visuospatial attention tasks. In a visuospatial sequence task [17], the amplitude of the P1 was used to measure the learning effect, and the P1 amplitude decreased significantly with repeated learning. This indicated a reduced demand for visual attentional resources. Another study argued that P2 was the ERP component associated with visual spatial attention. The higher the amplitude of P2, the more focused the attention was, which indicated that more attention resources were allocated [18]. The Event-Related Synchronization (ERS) and Event-Related Desynchronization (ERD) phenomena of the EEG rhythms theta and alpha have also been suggested to characterize the level of attention and allocation of attentional resources of the brain in visuospatial attention tasks. The more prominent theta-ERS and alpha-ERS represent the idler and more inattentive state of the brain [19,20]. In contrast, the more prominent alpha-ERD represents the increased excitability of cortical areas and neurons [21,22].

In conclusion, the above research only discussed subjects’ behavioral advantages when the target appeared in the upper and lower visual fields. However, the internal neurophysiological characteristics of the brain are not clear, and there is a lack of comprehensive analysis of the external behavior of the subjects and their internal neurophysiological characteristics. Therefore, we studied the effect of the target appearing in the upper and lower visual fields on the allocating attention resources in subjects’ brains in different tasks. We designed two different types of task paradigms: a spatial location orientation task paradigm related to “spatial location information” and a visual search task paradigm related to “cognition and recognition”. Our study used the mean amplitude of P1 component and the mean amplitude of P2 component during their latencies and the ERS and ERD phenomena of EEG rhythms theta and alpha as indicators of attention level and attentional resource allocation of the brain. We analyzed the external behavior (response accuracy and reaction time) of the subjects and the internal neurophysiological characteristics of their brains, as well as the relationship between them. The purpose is to provide a basis for the design of rehabilitation tasks for stroke patients with low attention levels, such as those with a spatial attention disorder. It is crucial for improving attentional function and recovering limb motor function in stroke patients [6,23,24,25].

## 2. Materials and Methods

### 2.1. Participants

Twenty healthy subjects participated in the current experiments. All subjects were right handed and had normal or corrected-to-normal vision. Subjects were recruited from the Cixi Institute of Biomedical Engineering, Ningbo Institute of Materials Technology and Engineering, Chinese Academy of Sciences. During the pre-processing analysis, data from two subjects were discarded because of bad EEG data quality (e.g., strong drifts and extensive muscle artifacts). As a result, the EEG data of 18 subjects (10 males and 8 females, mean age of 24.0 ± 2.0 years) have been retained. A priori analysis of the required sample size was conducted using G-Power. The sample size of 18 for two-tailed paired *t*-tests was estimated for an alpha error probability of 0.05, a power of 0.75, and an effect size of 0.66. For the two-factor repeated-measures Analyses of Variance (ANOVAs), this sample size was estimated for an alpha error probability of 0.05, a power of 0.93, and an effect size of 0.3. We conducted all experimental procedures according to the Declaration of Helsinki. Before the experiment, all subjects signed written informed consent approved by the local Research Ethics Committee.

### 2.2. Experimental Paradigms

**Figure 1:**
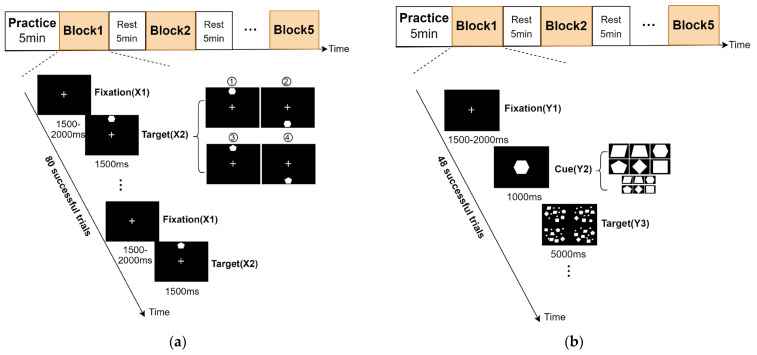
Experimental paradigms. (**a**) Spatial location orientation task paradigm. This experimental task paradigm contains five consecutive blocks, each block has 80 trials, and each trial contains two stimuli (X1 and X2). The specific explanation and experimental task procedures can be seen in Section 2.3. (**b**) Visual search task paradigm. This experimental task paradigm contains five consecutive blocks, each block has 48 trials, and each trial contains three stimuli (Y1, Y2, and Y3). More details of this paradigm can be seen in Section 2.3.

To investigate the overall trend of brain attention resource allocation advantages and behavioral performance advantages of subjects when the target appeared in the upper and lower visual fields in different tasks, we designed a spatial location orientation task paradigm and a visual search task paradigm. In the spatial location orientation task paradigm, we defined that the target appearing on the upper side of the screen’s central fixation point was “the target in the upper visual field” and the target appearing on the lower side of the screen’s central fixation point was “the target in the lower visual field”. In the visual search task paradigm, we defined that the target appearing on the upper left of the screen’s central fixation point was “the target in the upper left visual field”, the target appearing on the lower left of the screen’s central fixation point was “the target in the lower left visual field”, the target appearing on the upper right of the screen’s central fixation point was “the target in the upper right visual field”, and the target appearing on the lower right of the screen’s central fixation point was “the target in the lower right visual field”. In order to investigate the above overall trend, we set gaps in the four visual fields to distinguish stimuli distributed in the four visual fields. In the spatial location orientation task, the subjects needed to locate the target according to the target’s spatial location. In the visual search task, subjects needed to search for the target in the entire visual space. So, in our study, we allowed subjects to make slight eye movements during the experiments. As can be seen in Figure 1a (Experiment 1) and Figure 1b (Experiment 2), the subjects were required to complete Experiment 1 and then Experiment 2. The experiments were conducted in an electromagnetic shielding room to reduce environmental disturbance. Before the experiments, subjects were asked about their mood and physical condition and then began the experiments. The stimulus screen was two-dimensional, with a size of 19.5 inches. All stimuli were white and displayed on a black background. The subjects sat at a distance of 70 cm from the screen. Subjects were asked to make a key response with their right index and middle fingers and avoid body movements other than the right index and middle finger movements. Avoiding blinking and swallowing as much as possible was also required during the experiments. Before each experiment, there was a practice session of approximately five minutes to help subjects familiarize the task rules and keystroke specifications. Each experiment consisted of five consecutive blocks, each block contained 80 trials in Experiment 1 (48 trials in Experiment 2), and each block lasted for about five minutes. Note that since the single trial time was much shorter in Experiment 1, each experimental block took approximately the same amount of time. Before the start of each block, subjects were asked to adjust their sitting posture and position in line with the practice and fix the gaze point to the central fixation cross (“+”). Since the whole experiment was related to the upper and lower visual fields, it was necessary to adjust the monitor and seat height according to the subject’s height before the experiments. The purpose was to ensure that the middle position of the subject’s eyes was flush with the central fixed cross (“+”) in both horizontal and vertical directions. Between blocks, there was a programmed break of five minutes to avoid fatigue. After these breaks, subjects initiated the following block via a button press and were thus free to take longer breaks if needed. The recording session lasted approximately 60 min including breaks, so the two experiments lasted about 120 min.

### 2.3. Experiment Procedures

In Experiment 1, subjects were required to perform Task A. The entire experiment consisted of five consecutive blocks, each block containing 80 trials. Each trial contained two stimuli, the “+” stimulus (X1), which served as the preparatory stimulus and was presented for 1500 ms to 2000 ms. An imperative stimulus (X2) followed, in which a random pentagon or hexagon appeared above or below the central fixation cross. The hexagon was the target figure, and the pentagon was the non-target figure. The subjects’ task was to detect the position where the target figure appeared and ignore the non-target figure. The target figure and non-target figure appeared 40 times at random, and each appeared above and below the central fixation cross 20 times at random. The eccentricity of the target figure was 10.78°. The subjects pressed the “5” key if the target figure was detected above and the “2” key if it was below. The X2 disappeared while the button was pressed. If no key response were detected, X2 would last 1500–2000 ms, then the subject would proceed to the next trial. In Experiment 2, the subjects needed to perform Task B. The experiment consisted of five consecutive blocks, each block containing 48 trials. Each trial contained three stimuli. The “+” stimulus (Y1), which served as the preparatory stimulus, was presented for 1500 ms to 2000 ms. A cue stimulus (Y2) appeared after the Y1, indicating the target’s figure to be searched. After 1000 ms, the search stimulus (Y3) appeared. Y3 consisted of 12 kinds of figures (large square, small square, large rhombus, small rhombus, large trapezoid, small trapezoid, large hexagon, small hexagon, large parallelogram, small parallelogram, large pentagon, and small pentagon). The 12 kinds of figures appeared randomly in four quadrants, with 12 figures in each quadrant. Notably, the previous cued stimulus figure (target figure) appeared only in one of the four quadrants of the subsequent search stimulus, and 12 target figures appeared randomly once in each of the four quadrants (12 × 4 = 48). So, the Y3 contained one target figure and 47 non-target figures. The eccentricity range of the target figure was 2.78° to 17.94°. Subjects needed to find the location where the target figure appeared in Y3 within 5000 ms and press the key to respond. If the target figure appeared on the left, the subjects needed to press “4”, and if it appeared on the right, the subjects needed to press “6”. The Y3 disappeared while the button was pressed and the Y3 lasted 5000 ms if no key response was detected. Then, the subsequent trial took place.

### 2.4. Data Acquisition

The EEG data were recorded from Ag/AgCl electrodes using Synamps-2 amplifiers and Neuroscan acquisition software (Neuroscan, El Paso, TX, USA). These electrodes were mounted in a 64-channel electrode cap (Quick-Cap, Neuroscan, El Paso, TX, USA). Two additional monopolar electrodes were located at the left and right mastoids (M1, M2). Two bipolar electrode pairs were used in the present experiments. They were used for the vertical and horizontal electrooculogram (EOG) recording to detect eye movements and blinks. Electrodes were arranged according to the international 10–20 system. The sampling rate of the EEG was set to 1000 Hz. The impedance of each electrode was less than 10 kΩ for all. The 64-channel EEG data we collected covered the whole head.

### 2.5. Date Analysis

The 64-channel EEG data were processed offline using the CURRY 7 software package (CURRY Neuroimaging Suite 7.0.9 XS, Neuroscan, El Paso, TX, USA). The EEG continuous recordings were re-referenced offline by the average of two mastoids, applying the constant baseline correction and a 0.1–30 Hz bandpass filter. We analyzed the 64-channel EEG data, and statistically analyzed the EEG data results of the parieto-occipital region. We used the artifact removal method of CURRY 7 software to remove blink artifacts and bad blocks. The bad block mainly contained motion artifacts, such as body and head motion artifacts. Finally, artifacts-free EEG signals were retained with voltages between ±100 μV.

In Task-A, the EEG signals were segmented into 1200 ms epochs. Each epoch was defined as −200–1000 ms around the onset of X2. The 200 ms before the X2 display served as the baseline. In Task A, we defined “the upper visual field” as U and “the lower visual field” as D. So, the X2 contained four conditions. We defined “the target appeared in the upper visual field” as U_target, “the non-target appeared in the upper visual field” as U_non-target, “the target appeared in the lower visual field” as D_target, and “the non-target appeared in the lower visual field” as D_non-target. A parieto-occipital region of interest (ROI_1_) consisting of PO7, POZ, and PO8 was chosen for data analysis. This region, as well as the regions used in Task B, were chosen because they are associated with visual input, and they have been selected for data analysis in most previous studies of visuospatial selective attention [26,27,28]. Data analysis included three levels: behavior, time domain, and time-frequency domain. The behavioral analysis results were mainly reflected in the subjects’ response time and response accuracy. It was worth noting that since the average response time of subjects among all trials for the target that appeared in U and D was 644 ms, only EEG data within 644 ms from the onset of the stimulus were analyzed. In time-domain analysis, the EEG signals in the above four conditions were averaged separately over ROI_1_ across all trials for all subjects. For a more detailed analysis, the epochs were divided into three phases (0–200 ms as PhaseA-I, 200–400 ms as PhaseA-II, and 400–644 ms as PhaseA-III). We analyzed the differences in voltage amplitude and brain activation levels in the above four conditions. For time-frequency domain analysis, the short-time Fourier transform was performed separately from 1–30 Hz (in steps of 0.5 Hz) for each trial, with analysis windows centered every 200 ms. Then, we calculated the average power value of all trials for a single subject and averaged the results of all subjects. Based on the difference in the above four conditions, we defined 0–350 ms as the early stage of stimulus presentation and 350–644 ms as the late stage of stimulus presentation. Further analysis included three analysis levels, the theta-ERS (3–8 Hz) and alpha-ERS (8–12 Hz) phenomena in the early stage of stimulus presentation, and the alpha-ERD (8–12 Hz) phenomenon in the late stage of stimulus presentation We defined the above three analysis levels as PhaseA-theta-ERS, PhaseA-alpha-ERS, and PhaseA-alpha-ERD, respectively. Finally, the correlation between the internal neurophysiological results and the external behavioral results was summarized.

In Task-B, the EEG signals were divided into 3200 ms segments. Each segment was defined as −200–3000 ms around the onset of Y3. The 200 ms before the Y3 display served as the baseline. In Task-B, our analysis included two independent levels on both the left (L) and the right ^®^. We defined “the upper left visual field” as LU, “the lower left visual field” as LD, “the upper right visual field” as RU, and “the lower right visual field” as RD. For the target that appeared on the left, a region of interest (ROI_2_) consisting of left parieto-occipital electrodes PO3, PO5, and PO7 was chosen. Another region of interest (ROI_3_) consisting of right parieto-occipital electrodes PO4, PO6, and PO8 was chosen for data analysis for the target that appeared on the right [29]. Data analysis included three levels: behavior, time domain, and time-frequency domain. The behavioral analysis results were mainly reflected in the response time and response accuracy of the subjects. The average reaction time of subjects to the target stimulus was 2186 ms on the left and 2790 ms on the right. Therefore, only the EEG data within 2186 ms from the moment of the target stimulus appearance were analyzed for the stimuli on the left. For the right target, only the EEG data within 2790 ms from the moment of the target stimulus appearance were analyzed. In time-domain analysis, the EEG signals of the target that appeared in LU and LD were averaged separately over ROI_2_ across all trials for all subjects. The EEG signals of the target that appeared in RU and RD were averaged separately over ROI_3_ across all trials for all subjects. We only analyzed the ERP components of P1 and P2, because the differences between the target that appeared in LU and LD, as well as the differences between the target that appeared in RU and RD, were mainly manifested in them. In the current experiment, P1 and P2 components were determined as the mean amplitude within the time window from 80 ms to 130 ms and the time window from 200 ms to 400 ms, respectively. The selection of the latencies was determined by previous research on visual selective attention of P1 and P2 components, as well as our data results [18,30]. We defined PhaseB-P1 and PhaseB-P2 as the two analysis levels for the following analysis. We also defined 0–500 ms as the early stage of target presentation. For time-frequency domain analysis, the short-time Fourier transform was performed the same as Task-A. We defined 500–1000 ms as the medium stage of target presentation and 1000 ms later as the late stage of target presentation. For the target that appeared on the left, time-frequency domain analysis included three analysis levels, the theta-ERS (3–8 Hz) and alpha-ERD (10–14 Hz) phenomena in the medium stage of target presentation, and the alpha-ERS (10–14 Hz) phenomenon in the late stage of target presentation. We defined the above three analysis levels as PhaseB-theta-ERS, PhaseB-alpha-ERD, and PhaseB-alpha-ERS. For the target that appeared on the right, our analysis included three analysis levels. The theta-ERS (3–8 Hz) and alpha-ERD (10–14 Hz) phenomena in the medium stage of target presentation, and the alpha-ERD (10–14 Hz) phenomenon in the late stage of target presentation. We defined the above three analysis levels as PhaseB-theta-ERS, PhaseB-alpha-ERD_1_, and PhaseB-alpha-ERD_2_. Finally, the correlation between the internal neurophysiological results and the external behavioral results was summarized.

### 2.6. Statistical Analysis

Behavioral and EEG data were statistically analyzed using paired *t*-tests and repeated-measures Analyses of Variance with the statistical analysis software SPSS (version 23). Specifically, in Task A, we used paired *t*-tests to analyze the results of behavioral data at the different stimulus conditions (U_target, D_target), repeated-measures ANOVAs with factors of the stimulus condition (U_target, U_non-target, D_target, D_non-target) and the analysis level (PhaseA-I, PhaseA-II, PhaseA-III) for EEG data in time-domain results, and repeated-measures ANOVAs with factors of the stimulus condition (U_target, U_non-target, D_target, D_non-target) and the analysis level (PhaseA-theta-ERS, PhaseA-alpha-ERS, PhaseA-alpha-ERD) for EEG data in time-frequency domain results. In Task B, for the target on the left, paired *t*-tests was used to analyze the result of behavioral data at different visual field levels (LU vs. LD), repeated-measures ANOVAs with factors of the visual field (LU vs. LD) and the analysis level (PhaseB-P1, PhaseB-P2) were performed for EEG data in time-domain results, and two-way repeated-measures ANOVAs with factors of the visual field (LU vs. LD) and the analysis level (PhaseB-theta-ERS, PhaseB-alpha-ERD, PhaseB-alpha-ERS) was performed for EEG data in time-frequency domain results. For the target on the right, a paired *t*-test was used to analyze the result of behavioral data at different visual field levels (RU vs. RD), the repeated-measures ANOVAs with factors of the visual field (RU vs. RD) and the analysis level (PhaseB-P1, PhaseB-P2) was performed for EEG data in time-domain results, and two-way repeated-measures ANOVAs with factors of the visual field (RU vs. RD) and the analysis level (PhaseB-theta-ERS, PhaseB-alpha-ERD_1_, PhaseB-alpha-ERD_2_) was performed for EEG data in time-frequency domain results. Finally, *p* < 0.05 was considered statistically significant.

## 3. Results

### 3.1. Exp.1 Behavioral Results

In Task A, the presentation time of the target stimulus (X2) was 1500 ms. So, the key responses that exceeded 1500 ms (including correct and wrong responses) were considered as outliers and were excluded. The two-tailed paired *t*-tests were performed for the response time and response accuracy between U and D. The subjects’ response time for the target that appeared in D (Mean ± SD = 634 ± 127 ms) was faster than the target that appeared in U (Mean ± SD = 654 ± 128 ms). The result was statistically significant (*t*_(17)_ = 3.255, *p* = 0.005). However, the response accuracies of the subjects for the target that appeared in U (Mean ± SD = 0.996 ± 0.007) and D (Mean ± SD = 0.996 ± 0.008) were essentially perfect and so could not discriminate between them (*t*_(17)_ < 0.001, *p* = 1).

### 3.2. Exp.1 Time-Domain Results

**Figure 2:**
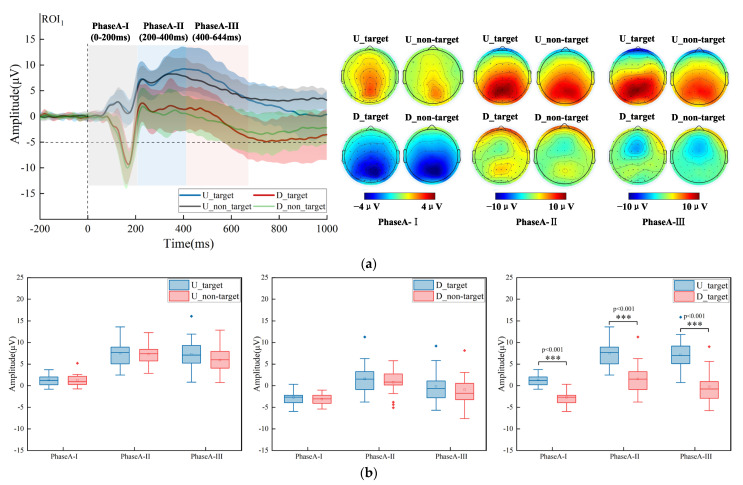
(**a**) Grand-average time-domain waveforms over ROI_1_ and the brain topographic maps in three analysis levels (PhaseA-I, PhaseA-II, PhaseA-III). (**b**) Statistical analysis results over ROI_1_ in three analysis levels (PhaseA-I, PhaseA-II, PhaseA-III). In each sub-graph, two different colors represent two different categories. The horizontal line represents the median, the small hollow rectangle represents the average value, and the solid diamond represents the outlier that deviates from the center. The p value represents significance level. (Note: *** *p* < 0.001.)

As seen from the group-averaged time-domain waveforms and topographic maps in Figure 2a, firstly, there were differences in ERP waveforms induced by target conditions (U_target, D_target) and non-target conditions (U_non-target, D_non-target). Compared with the target conditions, in PhaseA-I (U_target_Mean ± SD_ = 1.307 ± 1.200 μV, U_non-target_Mean ± SD_ = 1.239 ± 1.396 μV, D_target_Mean ± SD_ = −2.717 ± 1.651 μV, D_non-target_Mean ± SD_ = −3.111 ± 1.287 μV), the non-target conditions induced stronger negative ERP components and negative activation in the parieto-occipital region. In PhaseA-II (U_target_Mean ± SD_ = 7.528 ± 2.854 μV, U_non-target_Mean ± SD_ = 7.325 ± 2.357 μV, D_target_Mean ± SD_ = 1.558 ± 3.625 μV, D_non-target_Mean ± SD_ = 0.745 ± 2.965 μV) and PhaseA-III (U_target_Mean ± SD_ = 7.247 ± 3.885 μV, U_non-target_Mean ± SD_ = 5.912 ± 3.000 μV, D_target_Mean ± SD_ = −0.166 ± 3.822 μV, D_non-target_Mean ± SD_ = −0.942 ± 3.538 μV), the non-target conditions induced weaker positive ERP components and the positive activation of the parieto-occipital region was also weaker. A two-factor repeated-measures ANOVAs with factors of the stimulus condition (U_target, U_non-target) and the analysis level (PhaseA-I, PhaseA-II, PhaseA-III) was performed. The statistical analysis results are shown in Figure 2b. The interaction was not significant, and the main effect of the stimulus condition was not significant (F (1, 17) = 0.873, *p* = 0.363, η^2^ = 0.049). A two-factor repeated-measures ANOVAs with factors of the stimulus condition (D_target, D_non-target) and the analysis level (PhaseA-I, PhaseA-II, PhaseA-III) was performed. The interaction was not significant, and the main effect of the stimulus condition was not significant (F (1, 17) = 2.254, *p* = 0.152, η^2^ = 0.117). Secondly, the EEG signals induced by the conditions of U_target and D_target were highly variable. Compared to the U_target condition, in PhaseA-I, the D_target condition induced stronger negative ERP components and negative activation in the parieto-occipital region. In PhaseA-II and PhaseA-III, the D_target condition induced weaker positive ERP components, and the positive activation of the parieto-occipital region was also weaker. A two-way repeated-measures ANOVAs with factors of the stimulus condition (U_target, U_non-target) and the analysis level (PhaseA-I, PhaseA-II, PhaseA-III) over ROI_1_ was performed. The statistical analysis results were shown in Figure 2b. The interaction was not significant between the stimulus condition and the analysis level, but the main effect of the stimulus condition was significant (F (1,17) = 225.763, *p* < 0.001, η^2^ = 0.930). The simple effect analysis of the stimulus condition showed a significant effect over PhaseA-I (F (1, 17) = 200.888, *p* < 0.001, η^2^ = 0.922), PhaseA-II (F (1, 17) = 120.522, *p* < 0.001, η^2^ = 0.876), and PhaseA-III (F (1, 17) = 101.222, *p* < 0.001, η^2^ = 0.856). Therefore, in the above time-domain analysis results, the negative ERP component related to spatial orientation in PhaseA-I indicated the transient response of the brain to external stimuli and the stronger perceptual sensitivity of the dorsal striated cortex to the stimulus in D [31,32]. The subsequent positive ERP component in PhaseA-II and PhaseA-III showed that the process of the brain for the non-target stimulus consumed relatively fewer attention resources than the target stimulus [33]. Compared with the target stimulus in U, the subjects spent fewer attention resources on the target stimulus in D.

### 3.3. Exp.1 Time-Frequency Results

**Figure 3:**
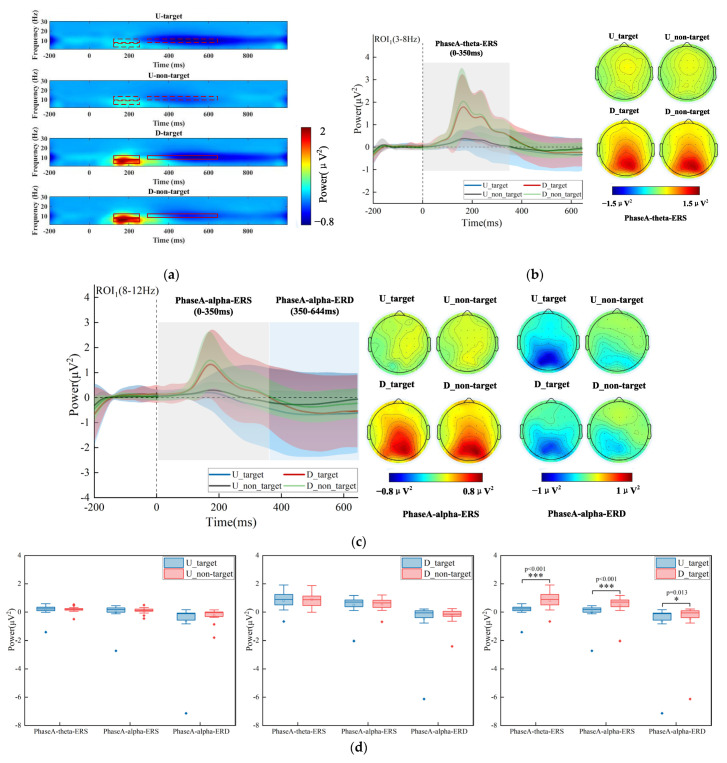
Time-frequency analysis results. (**a**) Group-averaged time-frequency spectrogram over ROI_1_, red and blue patterns indicate power values relative to the baseline. (**b**) Spectrogram of theta band that averaged over ROI_1_ and scalp distribution of theta power. (**c**) Spectrogram of alpha band that averaged over ROI_1_ and scalp distribution of alpha power. (**d**) Statistical analysis results over ROI_1_ in three analysis levels (PhaseA-theta-ERS, PhaseA-alpha-ERS, and PhaseA-alpha-ERD). In each sub-graph, two different colors represent two different categories. The horizontal line represents the median, the small hollow rectangle represents the average value, and the solid diamond represents the outlier that deviates from the center. The p value represents significance level. (Note: * *p* < 0.05, *** *p* < 0.001.)

The group-averaged time-frequency spectrogram in the whole frequency band (1–30 Hz) is shown in Figure 3a. In addition, we performed spectrum analyses separately on the theta band (3–8 Hz) and alpha band (8–12 Hz), as shown in Figure 3b,c. Firstly, there were differences in theta-ERS phenomenon (U_target_Mean ± SD_ = 0.147 ± 0.420 μV^2^, U_non-target_Mean ± SD_ = 0.188 ± 0.210 μV^2^, D_target_Mean ± SD_ = 0.818 ± 0.592 μV^2^, D_non-target_Mean ± SD_ = 0.886 ± 0.489 μV^2^), alpha-ERS phenomenon (U_target_Mean ± SD_ = 0.017 ± 0.702 μV^2^, U_non-target_Mean ± SD_ = 0.107 ± 0.212 μV^2^, D_target_Mean ± SD_ = 0.501 ± 0.706 μV^2^, D_non-target_Mean ± SD_ = 0.558 ± 0.437 μV^2^), and alpha-ERD phenomenon (U_target_Mean ± SD_ = −0.622 ± 1.651 μV^2^, U_non-target_Mean ± SD_ = −0.213 ± 0.463 μV^2^, D_target_Mean ± SD_ = −0.460 ± 1.444 μV^2^, D_non-target_Mean ± SD_ = −0.251 ± 0.582 μV^2^) between the target conditions (U_target, D_target) and the non-target conditions (U_non-target, D_non-target). Compared with the target conditions, non-target conditions induced stronger theta-ERS and alpha-ERS phenomena, and a weaker alpha-ERD phenomenon. The statistical analysis results are shown in Figure 3d. A two-factor repeated-measures ANOVAs with factors of the stimulus condition (U_target, U_non-target) and the analysis level (PhaseA-theta-ERS, PhaseA-alpha-ERS, PhaseA-alpha-ERD) was performed. The interaction was not significant, and the main effect of the stimulus condition was not significant (F (1, 17) = 0.628, *p* = 0.439, η^2^ = 0.036). A two-factor repeated-measures ANOVA with factors of the stimulus condition (D_target, D_non-target) and the analysis level (PhaseA-theta-ERS, PhaseA-alpha-ERS, PhaseA-alpha-ERD) was performed. The interaction and the main effect of the stimulus condition were not significant (F (1, 17) = 0.311, *p* = 0.585, η^2^ = 0.018). Secondly, compared to the U_target condition, The D_target condition induced stronger theta-ERS and alpha-ERS phenomena, and a weaker alpha-ERD phenomenon. The difference was mainly reflected in the parieto-occipital area. A two-way repeated-measures ANOVA with factors of the stimulus condition (U_target, D_target) and the analysis level (PhaseA-theta-ERS, PhaseA-alpha-ERS, PhaseA-alpha-ERD) over ROI_1_ was performed. The statistical analysis results are shown in Figure 3d. The interaction between the stimulus condition and the analysis level was not significant, but the main effect of the stimulus condition was significant (F (1, 17) = 68.082, *p* < 0.001, η^2^ = 0.800). The simple effect analysis of the stimulus condition showed a significant effect over PhaseA-theta-ERS (F (1, 17) = 56.757, *p* < 0.001, η^2^ = 0.770), PhaseA-alpha-ERS (F (1, 17) = 55.868, *p* < 0.001, η^2^ = 0.767), and PhaseA-alpha-ERD (F (1, 17) = 7.700, *p* = 0.013, η^2^ = 0.312). Therefore, the above time-frequency analysis results showed that compared with the target stimulus, subjects paid less attention to the non-target stimulus and spent fewer attention resources. Compared with the target stimulus in U, the brain processed the stimulus in D more easily and spent fewer attention resources.

So, based on the analysis results of behavior, time domain, and time-frequency domain, we summarized that in the process of visual perception, the stimulation in D induced the transient excitation of the brain. In visual processing, compared with the target stimulus, the process of the brain for the non-target stimulus consumed fewer attention resources. Compared with the target stimulus in U, the brain processed the target stimulus in D more easily and consumed fewer attention resources. Finally, better behavior was achieved.

### 3.4. Exp.2 Behavioral Results

In Task B, the presentation time of the target stimulus (Y3) was 5000 ms. So, the key responses exceeding 5000 ms (including correct and wrong responses) were considered as outliers and were excluded. The two-tailed paired *t*-tests were performed for the response time and response accuracy between LU and LD, and RU and RD. For behavioral results, on the left, when the target appeared in LU, the reaction time of the subjects (Mean ± SD = 1923 ± 424 ms) was significantly faster (*t*_(17)_ = −4.254, *p* = 0.001) than the target that appeared in LD (Mean ± SD = 2449 ± 432 ms). The response accuracies were not significantly different (*t*_(17)_ = 0.383 *p* = 0.706) between the target that appeared in LU (Mean ± SD = 0.869 ± 0.099) and LD (Mean ± SD = 0.863 ± 0.082). On the right, the reaction time of the subjects when the target appeared in RU (Mean ± SD = 2789 ± 411 ms) was almost the same as when the target appeared in RD (Mean ± SD = 2791 ± 408 ms); the difference was not significant (*t*_(17)_ = −0.032, *p* = 0.975). There was no significant difference (*t*_(17)_ = −1.769, *p* = 0.095) in the response accuracies of subjects between the target that appeared in RU (Mean ± SD = 0.763 ± 0.136) and RD (Mean ± SD = 0.797 ± 0.120).

### 3.5. Exp.2 Time-Domain Results

**Figure 4:**
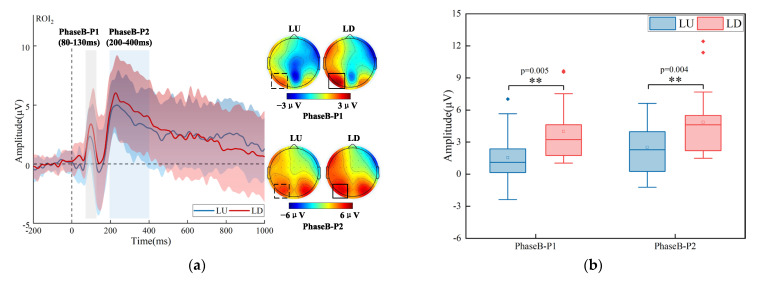
Time-domain analysis results for the target that appeared on the left. (**a**) Grand-average time-domain waveforms over ROI_2_ and brain topographic maps in two analysis levels (PhaseB-P1, PhaseB-P2). (**b**) Statistical analysis results over ROI_2_ in two analysis levels (PhaseB-P1, PhaseB-P2). The two different colors represent two different categories (LU and LD). The horizontal line represents the median, the small hollow rectangle represents the average value, and the solid diamond represents the outlier that deviates from the center. The p value represents significance level. (Note: ** *p* < 0.01.)

For the target on the left, the group-averaged time-domain waveforms (over ROI_2_) and brain topographic maps have been shown in Figure 4a. The amplitudes of P1 (LU_Mean ± SD_ = 1.531 ± 2.418 μV, LD_Mean ± SD_ = 2.511 ± 2.471 μV) and P2 (LU_Mean ± SD_ = 3.997 ± 2.703 μV, LD_Mean ± SD_ = 4.849 ± 3.121 μV) for the target that appeared in LU were lower than that in LD. The differences were mainly in the left parieto-occipital region. A two-way repeated-measures ANOVA with factors of the visual field (LU vs. LD) and the analysis level (PhaseB-P1, PhaseB-P2) over ROI_2_ was performed. The results can be seen in Figure 4b; the interaction between the visual field and the analysis level was not significant, but there was a significant main effect of the visual field (F (1, 17) = 15.621, *p* = 0.001, η^2^ = 0.479). The simple effect analysis of the visual field showed a significant effect over PhaseB-P1 (F (1, 17) = 10.302, *p* = 0.005, η^2^ = 0.377), PhaseB-P2 (F (1, 17) = 11.360, *p* = 0.004, η^2^ = 0.401).

**Figure 5:**
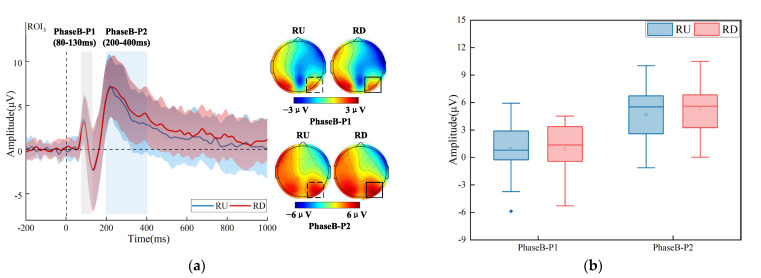
Time-domain analysis results for the target that appeared on the right. (**a**) Grand-average time-domain waveforms over ROI_3_ and brain topographic maps in two analysis levels (PhaseB-P1, PhaseB-P2). (**b**) Statistical analysis results over ROI_3_ in two analysis levels (PhaseB-P1, PhaseB-P2). The two different colors represent two different categories (RU and RD). The horizontal line represents the median, the small hollow rectangle represents the average value, and the solid diamond represents the outlier that deviates from the center.

For the target on the right, the time-domain waveforms and brain topographic maps have been shown in Figure 5a. The average amplitude of the P1 (RU_Mean ± SD_ = 0.898 ± 2.722 μV, RD_Mean ± SD_ = 0.901 ± 2.983 μV) was almost equal when the target appeared in RU and RD. The average amplitude of the P2 (RU_Mean ± SD_ = 4.679 ± 3.304 μV, RD_Mean ± SD_ = 5.292 ± 3.132 μV) was slightly lower when the target appeared in RU compared with the target that appeared in RD. A two-way repeated-measures ANOVA with factors of the visual field (RU vs. RD) and the analysis level (PhaseB-P1, PhaseB-P2) over ROI_3_ was performed. The results in Figure 5b showed that the interaction between the visual field and the analysis level was not significant. The main effect of the visual field was also not significant (F (1, 17) = 1.995, *p* = 0.176, η^2^ = 0.105).

Therefore, the results of time-domain analysis indicated that on the left, subjects were less attentive and needed fewer attentional resources in the early stage of the target that appeared in LU. On the right, there was no significant difference in the attention level and attentional resource consumption of the brain between the target that appeared in RU and RD.

### 3.6. Exp.2 Time-Frequency Results

**Figure 6:**
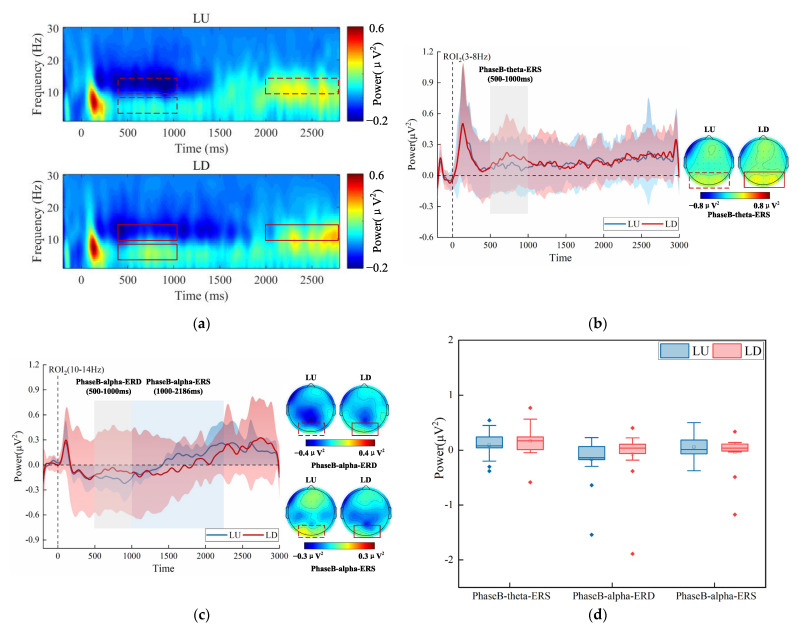
Time-frequency analysis results for the target that appeared on the left. (**a**) Group-averaged time-frequency spectrogram over ROI_2_, red and blue patterns indicate power values relative to the baseline. (**b**) Spectrogram of theta band (3–8 Hz) that averaged over ROI_2_ and scalp distribution of theta power. (**c**) Spectrogram of alpha band (10–14 Hz) that averaged over ROI_2_ and scalp distribution of alpha power. (**d**) Statistical analysis results over ROI_2_ in three analysis levels (PhaseB-theta-ERS, PhaseB-alpha-ERD, PhaseB-alpha-ERS). The two different colors represent two different categories (LU and LD). The horizontal line represents the median, the small hollow rectangle represents the average value, and the solid diamond represents the outlier that deviates from the center.

The group-averaged time-frequency spectrogram in the whole frequency band (1–30 Hz) was shown in Figure 6a. In addition, we performed spectrum analyses separately on the theta band (3–8 Hz) and alpha band (10–14 Hz), as shown in Figure 6b,c. In the medium stage of target presentation, the theta-ERS was weaker (LU_Mean ± SD_ = 0.089 ± 0.236 μV^2^, LD_Mean ± SD_ = 0.163 ± 0.278 μV^2^), and the alpha-ERD was stronger (LU_Mean ± SD_ = −0.168 ± 0.395 μV^2^, LD_Mean ± SD_ = −0.076 ± 0.481 μV^2^) in LU. The alpha-ERS was stronger (LU_Mean ± SD_ = 0.057 ± 0.228 μV^2^, LD_Mean ± SD_ = −0.036 ± 0.324 μV^2^) in LU in the late stage of target presentation. The differences mainly manifested in the parieto-occipital region. Figure 6d shows the statistical analysis results over ROI_2_. The interaction between the visual field (LU vs. LD) and the analysis level (PhaseB-theta-ERS, PhaseB-alpha-ERD, PhaseB-alpha-ERS) was not significant, and the main effect of the visual field was not significant (F (1, 17) = 0.265, *p* = 0.614, η^2^ = 0.015).

**Figure 7:**
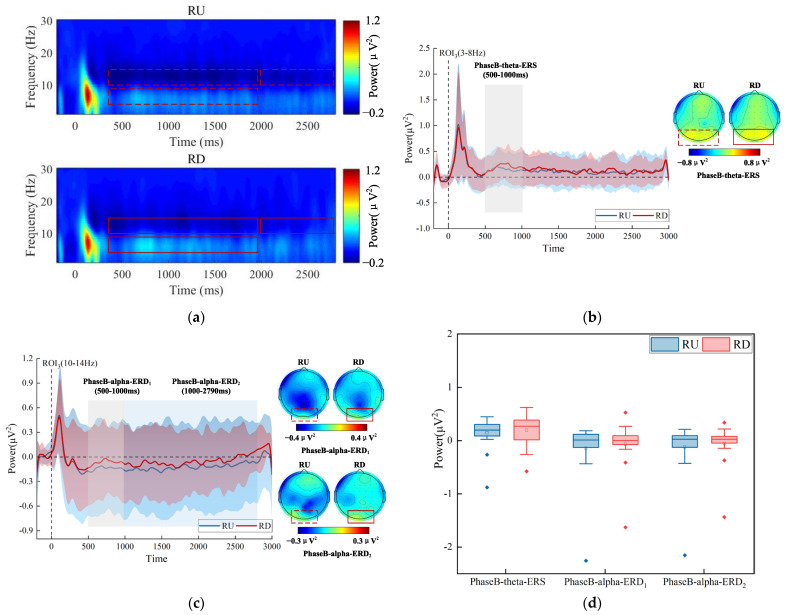
Time-frequency analysis results for the target that appeared on the right. (**a**) Group-averaged time-frequency spectrogram over ROI_3_, red and blue patterns indicate power values relative to the baseline. (**b**) Spectrogram of theta band (3–8 Hz) over ROI_3_ and scalp distribution of theta power. (**c**) Spectrogram of the alpha band (10–14 Hz) over ROI_3_ and scalp distribution of alpha power. (**d**) Statistical analysis results over ROI_3_ in three analysis levels (PhaseB-theta-ERS, PhaseB-alpha-ERD_1_, PhaseB-alpha-ERD_2_). The two different colors represent two different categories (RU and RD). The horizontal line represents the median, the small hollow rectangle represents the average value, and the solid diamond represents the outlier that deviates from the center.

For the target that appeared on the right, The group-averaged time-frequency spectrogram in the whole frequency band (1–30 Hz) is shown in Figure 7a. In addition, we performed spectrum analyses separately on the theta band (3–8 Hz) and the alpha band (10–14 Hz), as shown in Figure 7b,c. In the medium stage of target presentation, the theta-ERS was weaker (RU_Mean ± SD_ = 0.146 ± 0.307 μV^2^, RD_Mean ± SD_ = 0.194 ± 0.286 μV^2^), and alpha-ERD was stronger (RU_Mean ± SD_ = −0.141 ± 0.551 μV^2^, RD_Mean ± SD_ = −0.067 ± 0.434 μV^2^) in RU than in RD. The alpha-ERD was stronger (RU_Mean ± SD_ = −0.123 ± 0.529 μV^2^, RD_Mean ± SD_ = −0.057 ± 0.375 μV^2^) in RU in the late stage of target presentation. The differences mainly manifested in the parieto-occipital region. A two-way repeated-measures ANOVA with factors of the visual field (RU vs. RD) and the analysis level (PhaseB-theta-ERS, PhaseB-alpha-ERD_1_, PhaseB-alpha-ERD_2_) over ROI_3_ was performed. Figure 7d suggests that the differences between the visual field and analysis level were not significant.

Therefore, the results of time-frequency analysis indicated that on the left side, the attention level was higher in the medium stage when the target appeared in LU, but the attention level was lower in the late stage for the target that appeared in LU. However, on the right, the attention level was higher during the medium and late stages of the target that appeared in RU.

So, in Task-B, on the left, compared with the target that appeared in LD, the subjects could better mobilize attention resources and then demonstrated better behavior when the target appeared in LU. On the right, there was no significant difference in attention resource consumption and behavior when the target appeared in RU and RD.

## 4. Discussion

Asymmetries in human visual information processing have been an interesting topic in cognitive psychology and neuroscience. Most studies were based on left–right visual field asymmetries. These asymmetries were thought to reflect the functional differences between the left and right hemispheres [34,35,36,37,38]. Only a few studies were related to the asymmetry of the upper and lower visual fields. So, we designed two different task paradigms to investigate the neurophysiological asymmetry of the target in the upper and lower visual fields. The results showed that the asymmetry of the target appearing in the upper and lower visual fields was not only reflected in the behavior, but also in the neurophysiological characteristics of the brain. This provided new neurophysiological guidance for the design of rehabilitation tasks based on the upper and lower visual fields for stroke patients with a low level of attention.

Specifically, in Task A, behaviorally, the subjects’ reaction time was shorter when the target appeared in the lower visual field, which was consistent with a study on a spatial relocation memory task [10]. At the neurophysiological level, time-domain analysis results showed that when the target appeared in the lower visual field, the brain had a transient excitation in visual perception, and the brain consumed fewer attention resources during visual processing. For time-frequency analysis, our results showed that, when the target appeared in the lower visual field, the theta-ERS and alpha-ERS were stronger, and the alpha-ERD was weaker in the parieto-occipital region. This indicated that the brain processed more easily and expended fewer attentional resources for the target that appeared in the lower visual field. Previous literature has also suggested that more pronounced theta-ERS and alpha-ERS represented an idler and less focused state of the brain and more pronounced alpha-ERD represented the brain in a more concentrated state [39,40]. Therefore, for simple spatial position orientation tasks such as Task A, the brain can process visual information more efficiently when the target appears in the lower visual field. From the reaction time of the subjects to Task A, we can also find that the brain may only perform a simple visual processing process after receiving the input of visual information. Furthermore, we should note that the elicitation of ERP components and the interpretation of their neurophysiological significance depend on different task paradigms and visual stimuli. So, in the spatial location orientation task paradigm, we considered that this negative ERP component indicated the brain’s transient excitation caused by external stimuli, which was consistent with the previous study [31].

In Task B, on the left, behavior results show that when the target appeared in the upper left visual field, the reaction time of the subjects was significantly faster. The result was inconsistent with previous findings [11]. This difference may include the following reasons. One was, the visual search task paradigm was based on three-dimensional space in previous research, while our study was based on the two-dimensional plane. There will be depth differences of stimuli in three-dimensional space, while the stimulation in the two-dimensional plane will not have such differences. The differences would affect the behavior performance of the subjects in the search task. In addition, there was no “gap” among the stimuli of the four visual fields during the spatial search of previous research, while we set “gaps” among the four visual fields to distinguish the stimuli distributed in the different visual fields during the plane search of our study. This is consistent with the presentation of modern Chinese characters. So, most subjects searched from the upper left corner in the modern way of reading Chinese characters, in the order from left to right and from top to bottom. Therefore, the above two points may contribute to the advantage of the upper left visual field in the visual search task based on the plane in the current study. At the neurophysiological level, in the early stage of target presentation, we focused on the amplitudes of P1 and P2. Our results showed that the amplitudes of P1 and P2 were lower when the target appeared in the upper left visual field compared with the target in the lower left visual field. This suggested that the subjects had lower attention levels and consumed fewer attention resources when the target appeared in the upper left visual field. This was mainly manifested in the left parieto-occipital area. In the medium stage of target presentation, the theta-ERS was weaker, and the alpha-ERD was stronger when the target appeared in the upper left visual field. This indicated that more attention resources were consumed. However, in the late stage of target presentation, alpha-ERS appeared and was more pronounced when the target appeared in the upper left visual field, indicating that fewer attentional resources were consumed. This showed that during the whole stage of target presentation in Task-B, compared with the target that appeared in the lower left visual field, when the target appeared in the upper left visual field, the consumption of attention resources had a process from less to more and then to less, which reflected the process of brain attention resource mobilization. Therefore, for more complex visual search tasks such as Task B, the brain can process visual information more efficiently when the target appears in the upper visual field. From the reaction time of the subjects in Task B, it can be seen that compared with the simple spatial position orientation task, the brain has a more complex visual processing process for complex visual search tasks after receiving the input of visual information. That requires reasonable mobilization of attention resources to achieve better behavior. On the right, in our research results, when the target appeared in the upper right and lower right visual fields, there was no significant difference in the subject’s behavior and the consumption of brain attention resources. It is worth noting that the brain topographic maps of the alpha band showed an obvious alpha-ERD phenomenon in the sensorimotor region of the brain. This indicated that after receiving the input of visual information in the parieto-occipital region, the further processing of visual information transited to the sensorimotor region. This information transmission across brain regions is worthy of further analysis in subsequent studies. In addition, we should note that there were differences in the allocation of brain attention resources between the upper visual field and lower visual field in Task-B, but the differences were not significant. The EEG data results may be affected to some extent by eye artifacts (such as eye movement artifacts). Therefore, further work will use ICA to remove eye movement artifacts in EEG data, which may contribute to the consistency of EEG data results.

Overall, when the target appeared in the lower visual field in the spatial position orientation task, the subjects consumed fewer attention resources and displayed better behavioral performance. This indicated that the visual perception and processing were better for the target in the lower visual field in the spatial location orientation task. We can also see that better behavioral performance reflected higher processing efficiency [41]. Therefore, our results showed that the subjects reduced their brain attention load on the target in the lower visual field, thus improving the processing efficiency of the target in the lower visual field. In the visual search task, when the target appeared in the upper left visual field, the subjects’ brain attention resource consumption showed a process from less to more and then to less, and achieved better performance. This indicated that the visual perception and processing were better for the target in the upper left visual field in the visual search task. So, our results showed that the subjects could better mobilize attention resources and reduce their brain attention load on the target in the upper visual field during the visual processing, thus improving the processing efficiency of the target in the upper visual field.

**Figure 8:**
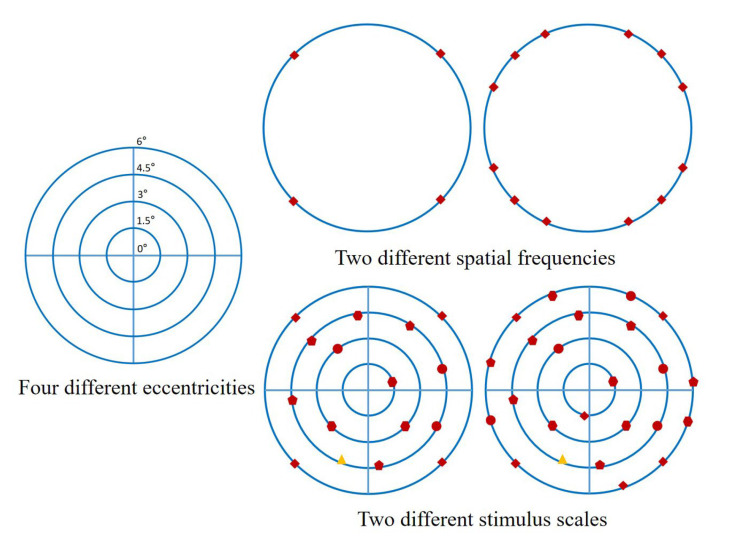
A preliminary experimental paradigm for refining different visual parameters of targets in different visual fields. The left side of the figure showed four different eccentricities of stimulus (1.5°, 3°, 4.5°, 6°). On the right side of the figure, the above shows the two different spatial frequencies of the stimulus (4, 12), and the below shows the two different scales of the stimulus (16, 24). Among them, the yellow graph is the target stimulus, and the other red graphs are the distractors.

Our study concluded the overall trend of attention advantage and behavior performance advantage of the human upper and lower visual fields in different tasks. For most stroke patients, their ventral-dorsal attention pathway is abnormal and their attention level is low. So, compared with healthy people, their perception and processing of task targets in the upper and lower visual fields are poor. Therefore, they need to make full use of the advantages of the upper and lower visual fields in different tasks to better perceive and process the task targets under the limited attention resources. Further research will extend to stroke patients and optimize the experimental paradigm on the basis of the study of upper and lower visual fields, combined with the characteristics of asymmetry of the left and right visual fields of stroke patients (such as hemianopia and neglect symptoms). At the same time, because the perception and processing of task targets in stroke patients are affected by more visual factors, the different visual parameters of the target stimulus (eccentricity, spatial frequency, stimulation scale, etc.) were refined. On the basis of the research on the visual parameter refinement of the target in the lower visual field (upper visual field), the research on visual parameter refinement of the left lower visual field and the right lower visual field (upper left visual field and upper right visual field) will be carried out. As can be seen in Figure 8, we refined the visual parameters of targets in different visual fields. The purpose was to investigate the effect of targets with different visual parameters in different visual fields on brain attention resource allocation and behavior performance of patients. In this way, we can further clarify the design principles of stroke rehabilitation tasks from a more detailed perspective. This also provides ideas for promoting the plasticity of the attention pathway in stroke patients.

## 5. Conclusions

In general, our study investigated the asymmetry and dominance of the target in the upper and lower visual fields in different tasks from the perspective of visual attention. The results showed that asymmetry and dominance are inseparable from behavior and neurophysiology. In the spatial position orientation task, when the target appeared in the lower visual field, the subjects consumed fewer attention resources and demonstrated better behavioral performance. In the visual search task, on the left, compared with the target that appeared in the lower left visual field, when the target appeared in the upper left visual field, the subjects can mobilize more attention resources to perform visual processing and achieve better behavior. Therefore, for people with low attention levels and insufficient allocation of attention resources, the spatial location orientation task and visual search task can be designed in the lower and upper visual fields, respectively. It is meaningful to promote the recovery of cognitive and motor functions of stroke patients with low attention levels.

## Data Availability

The data presented in this study are available on request from the corresponding author.
